# Left Radial Access Is Preferable to Right Radial Access for the Diagnostic or Interventional Coronary Procedures: A Meta-Analysis Involving 22 Randomized Clinical Trials and 10287 Patients

**DOI:** 10.1371/journal.pone.0078499

**Published:** 2013-11-05

**Authors:** Xiaogang Guo, Jie Ding, Yue Qi, Nan Jia, Shaoli Chu, Jinxiu Lin, Jinzi Su, Feng Peng, Wenquan Niu

**Affiliations:** 1 Department of Cardiology, The First Affiliated Hospital, School of Medicine, Zhejiang University, Hangzhou, China; 2 Department of Epidemiology, Beijing An Zhen Hospital, Capital Medical University, Beijing Institute of Heart, Lung and Blood Vessel Diseases, Beijing, China; 3 Department of Hypertension, Ruijin Hospital, School of Medicine, Shanghai Jiao Tong University, Shanghai, China; 4 Shanghai Institute of Hypertension, Ruijin Hospital, School of Medicine, Shanghai Jiao Tong University, Shanghai, China; 5 Department of Cardiology, The First Affiliated Hospital of Fujian Medical University, Fuzhou, China; 6 State Key Laboratory of Medical Genomics, Ruijin Hospital, School of Medicine, Shanghai Jiao Tong University, Shanghai, China; Washington Hospital Center, United States of American

## Abstract

**Objective:**

The transradial approach has been used extensively for both diagnostic and interventional coronary procedures; however, there is no universal consensus hitherto on the optimal choice of radial access from either the left or the right artery. We therefore sought to meta-analyze available randomized clinical trials to compare the left with the right radial access for the diagnostic or interventional coronary procedures.

**Methods and Results:**

Four electronic databases including the PubMed, EMBASE, Wanfang, and CNKI were searched up to April 2013. In total, there were 22 qualified randomized trials involving 5317 and 4970 patients assigned to the left and the right radial accesses, respectively. Data were extracted independently by two investigators. Analyses of the full data set indicated significant reductions in fluoroscopy time (seconds) (weighted mean difference; 95% confidence interval; P: −36.18; −53.28 to −18.53; <0.0005) and contrast use (mL) (−2.88; −5.41 to −0.34; 0.026) in patients with the left radial access compared to those with the right radial access, and there was strong evidence of heterogeneity but low probability of publication bias. The failure rate of radial access from the left was relatively lower than that from the right (odds ratio: 0.83; 95% confidence interval: 0.68−1.01; P = 0.064). Further in meta-regression analyses, body mass index was found to be a potential source of heterogeneity for both fluoroscopy time (regression coefficient: 35.85; P = 0.025) and catheter number (regression coefficient: 0.35; P = 0.018).

**Conclusions:**

Our findings demonstrate that left radial access is preferable to right radial access in terms of fluoroscopy time and contrast use for the diagnostic or interventional coronary procedures. The import of this study lies in its great shock to the concept of convenient radial access from the right artery.

## Introduction

The transradial approach has been used extensively for both diagnostic and interventional coronary procedures; however, there is no universal consensus hitherto on the optimal choice of radial access from either the left or the right artery [1]–[3]. Currently, this choice is largely dependent on the operator’s preference. The right radial access is generally preferred in routine clinical practice mainly due to its easier catheter manipulation for the operators from patient’s right side, and the current design of radial compression devices for the right wrist in medical market. As such, a major barrier to prevent the wide adoption of the left radial access lies in some difficulty to reach the left wrist leaning over the patient, particularly for shorter operators or in obese patients [4]. However, a great deal of attention has been recently directed toward the left radial access, as it has an important anatomical advantage due to the vascular anatomy of epiaortic vessels with a straighter route to the left coronary ostium, which could also reduce the risk of cerebrovascular complications [4]–[6]. The catheters in the right radial access, by contrast, must be rotated to afford the S-shaped geometry of the subclavian-innominate-aorta axis [4]. Although clinicians have conducted exhaustive research regarding the comparative efficacy and safety between the left and the right radial accesses, there is still no conclusive evidence. Some studies have documented that the right radial access was associated with shorter procedure time and lower incidence of access-site complications compared with the left radial access [7]. Contrastingly, a growing body of clinical trials have recently emerged to propose that radial access from the left artery might confer similar or even better procedural efficacy than from the right artery [6], [8], [9]. Literature, being abundant with such clinical trials, paves the way to identify whichever artery is the optimal choice for radial access; however, a comprehensive evaluation on this topic so far is lacking. To shed some light on this issue, we sought to summarize available randomized clinical trials to compare the left with the right radial access for the diagnostic or interventional coronary procedures via a meta-analysis.

## Methods

This meta-analysis of randomized clinical trials was carried out in accordance with the guidelines set forth by the Preferred Reporting Items for Systematic Reviews and Meta-analyses (PRISMA) statement (please see Checklist S1) [10].

### Search strategy

Four electronic databases including the PubMed, EMBASE (Excerpta Medica Database), Wanfang (http://www.wanfangdata.com.cn), and CNKI (China National Knowledge Infrastructure, http://www.cnki.net) were searched up to April 2013. The following subject terms were used: “right” AND “left” AND (“radial” OR “transradial”) AND (“coronary” OR “myocardial” OR “coronary syndrome” OR “intervention” OR “angiography”) AND (“study” OR “trial” OR “randomized” OR “randomised”). The search was supplemented by perusal of the bibliographies of retrieved papers and review articles, and by corresponding with the authors. Searching results were restricted to clinical trials and English or Chinese language.

### Trial selection

Abstracts of retrieved articles were independently read by two investigators (X.G. and F.P.) to assess their potential eligibility for this meta-analysis. When more than one publication of a trial existed, we abstracted the data from the most recent or the most complete publication. If necessary, we emailed the contributing authors to avoid the double counting of patients recruited in more than one trial by the same group. If a given trial can be split into two or more independent studies according to the purpose of cardiac catheterization, *viz*. either diagnostic or intervention coronary procedure, each was treated separately.

### Inclusion/exclusion criteria

For inclusion, trials had to be conducted in a randomized manner and compare the left with the right radial access for the diagnostic or interventional coronary procedures. Trials were excluded if they were cross-over trials or if they were conference abstracts, case reports, case series, editorials, review articles, or the non-English and non-Chinese articles.

### Data extraction

Two investigators (X.G. and F.P.) independently extracted data using a standardized Excel template (Microsoft Corp, Redmond, WA). Disagreements were resolved by consensus or by a third investigator (J.L.).

Data were collected on the first author, year of publication, ethnicity of the trial patients, sample size of each arm, fluoroscopy time (seconds), contrast use (mL), the number of catheters used, procedure time (minutes), the success rate of cardiac catheterization, dose-area product, and the percentages of radial spasm, radial hematoma, radial tortuosity, and subclavian tortuosity between the two arms, as well as the characteristics of trial patients, if available, including age, gender, body mass index (BMI), creatinine, and the percentages of smoking, hypertension, diabetes, dyslipidemia, prior percutaneous transluminal coronary angioplasty (PTCA), and prior myocardial infarction. Diagnosis of hypertension was based on the presence of elevated systolic (≥140 mmHg) and/or diastolic (≥90 mmHg) blood pressure and/or the current use of antihypertensive medications. Diabetes was diagnosed as fasting plasma glucose levels ≥7.0 mmol/L and/or non-fasting plasma glucose levels ≥11.0 mmol/L and/or taking hypoglycemic drugs or receiving parenteral insulin therapy.

### Statistical analyses

A meta-analysis was carried out with the available data from three or more independent studies for a certain outcome. Quantitative variables were summarized and compared by the weighted mean difference (WMD) with its 95% confidence interval (95% CI) between the patients with the left and the right radial accesses. Categorical variables were compared between the two group patients by the weighted odds ratio (OR) and its 95% CI under random-effects model.

Pearson correlation analyses were used to test the relationship between variables. The random-effects model using the DerSimonian & Laird method [11] was adopted irrespective of between-trial heterogeneity. Heterogeneity was assessed by χ^2^ test, and was quantified using the inconsistency index (*I* ^2^) statistic, which ranges from 0% to 100% and is defined as the percentage of the observed between-trial variability that is due to heterogeneity rather than chance.

Predefined subgroup analyses were conducted a priori according to the ethnicity of trial patients (mainly Caucasians and Asians), the purpose of cardiac catheterization (the diagnostic and interventional coronary procedures), and the identity of the operators (mainly experts and the mixture of experts and operators in training).

Influential analyses were performed to assess the contribution of individual trials to pooled effect estimates by sequentially omitting each trial one at a time and computing differential estimates for remaining trials. Meta-regression analyses were conducted to evaluate the extent to which different trial-level variables, including all characteristics of trial patients as mentioned above, explained the heterogeneity of pooled effects of coronary procedures on the outcomes examined.

Publication bias was evaluated by the Begg’s and Egger’s tests. The trim and fill method was also adopted to estimate the number and outcomes of potentially missing trials resulting from publication bias. P<0.05 was considered statistically significant with the exceptions of the *I* ^2^, Begg’s and Egger’s statistics, for which a significance level was defined as P<0.10 [12]. Data management and statistical analyses were conducted using STATA software (StataCorp, College Station, TX, version 11.2 for Windows).

## Results

### Eligible trials

Characteristics of the trial patients in this meta-analysis are presented in Table S1. The initial search for the randomized clinical trials that compared the left with the right radial access for the diagnostic or interventional coronary procedures yielded 435 potentially relevant articles. Applying further inclusion/exclusion criteria left a total of 22 articles [1], [2], [4]–[9], [13]–[26] involving 5317 patients assigned to the left radial access and 4970 patients assigned to the right radial access in final analyses. All qualified articles were published between 2001 and 2013, and ten of them were written in Chinese language [5], [18]–[26].

**Figure 1:**
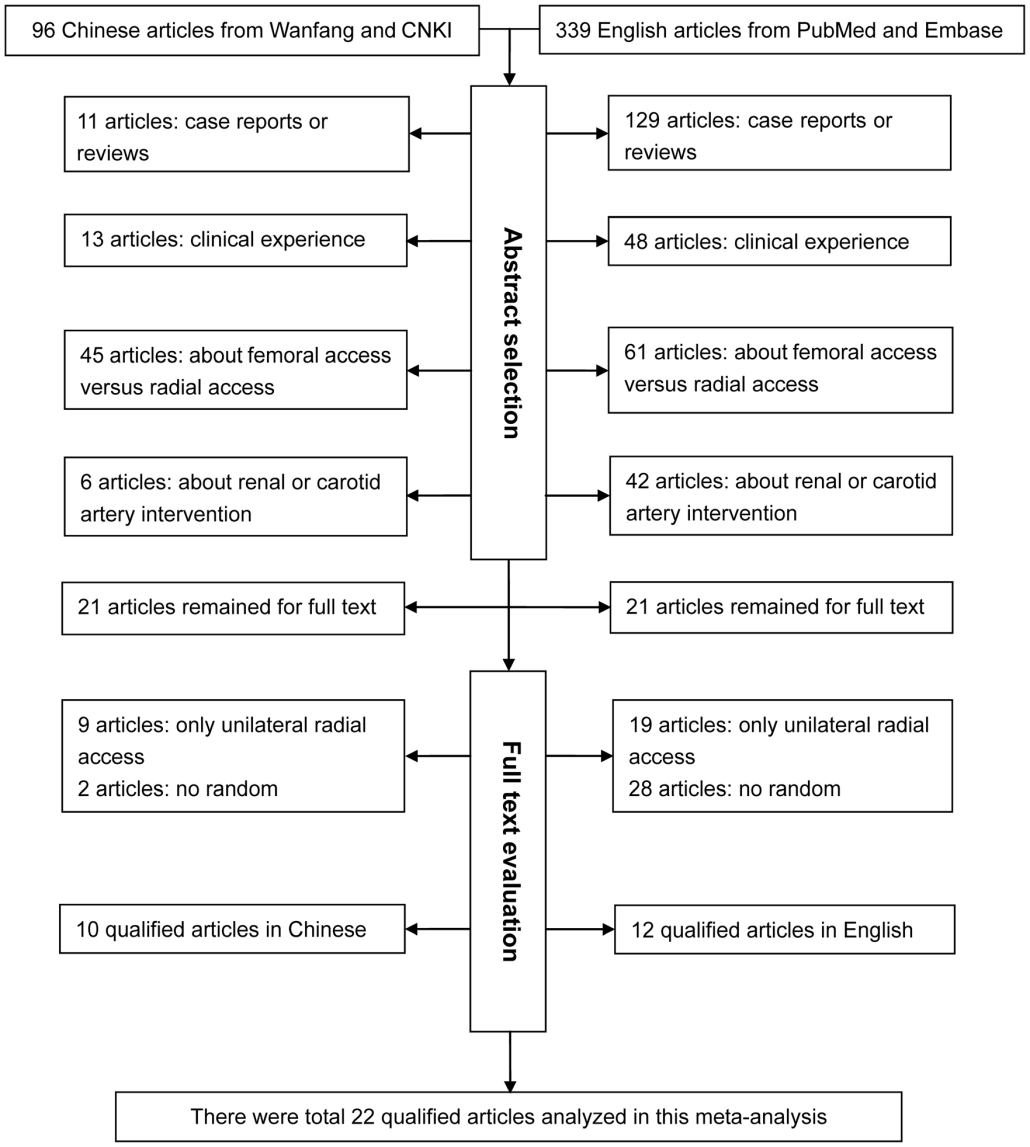
Flow diagram of search strategy and study selection.

A flow diagram schematizing the selection process of recruited articles with specific reasons is illustrated in Figure 1. Four of 22 qualified articles recorded outcomes separately by the diagnostic and interventional coronary procedures [4], [7], [18], [24], and one article additionally recorded outcomes from experts [9]. Nine articles involved patients of Caucasian descent [1], [4], [6], [9], [13]–[17], 12 articles of Asian descent [2], [5], [8], [18]–[26], and one article of mixed descents [7]. The average success rate of cardiac catheterization was 96.22% and 95.39% for the left and the right radial accesses, respectively (Table S1). The distributions of average age, gender, BMI, smoking, and the percentage of prior myocardial infarction were comparable between the patients with the left and the right radial accesses. Although the percentages of hypertension, diabetes and prior PTCA were slightly higher in patients with the left radial access than those with the right radial access, no statistical significance was reached.

There were no differences in the percentages of radial spasm, radial hematoma, and radial tortuosity between the patients with the left and the right radial accesses. The percentage of subclavian tortuosity was significantly higher in patients assigned to the right radial access (12.96%) than to the left radial access (2.78%) (P = 0.028).

### Overall analyses

**Figure 2:**
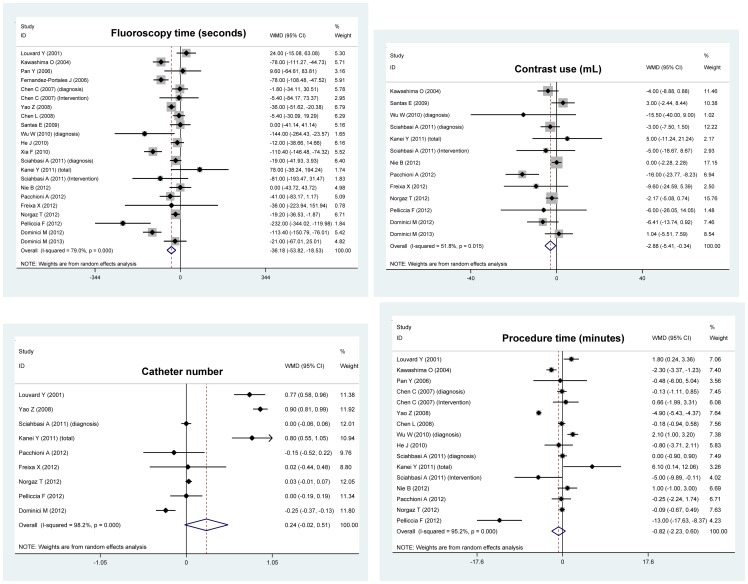
Forest plots of changes of fluoroscopy time, contrast use, catheter number, and procedure time for comparison of the left radial access with the right radial access.

**Figure 3:**
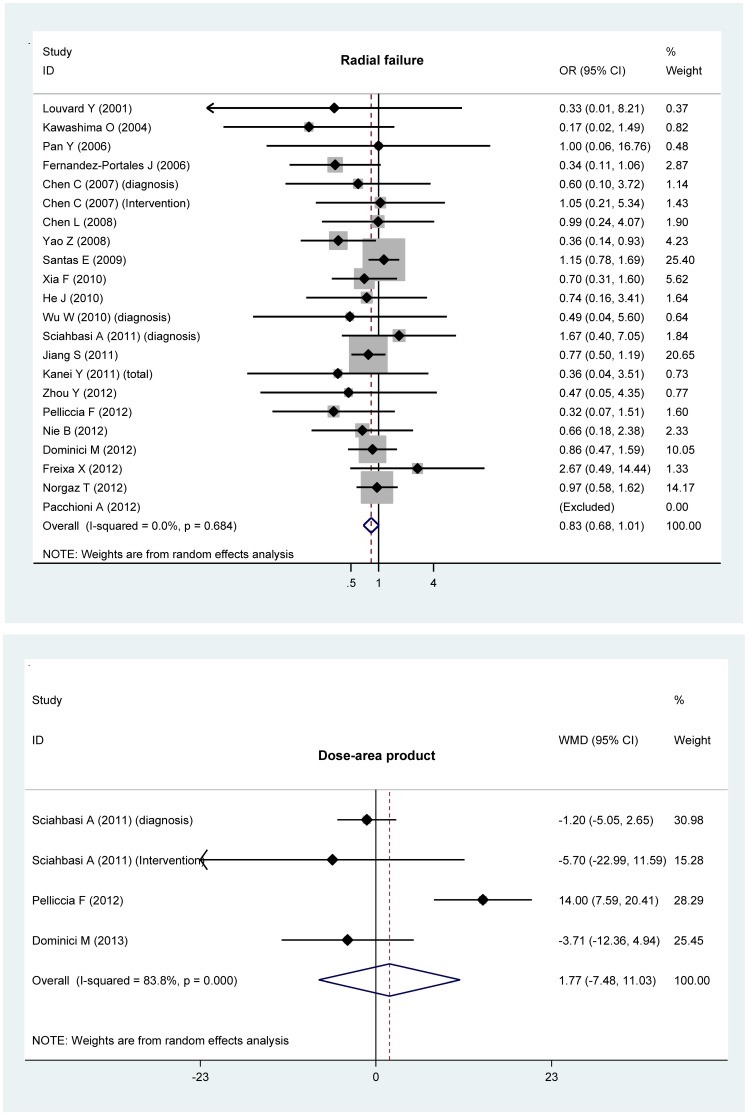
Forest plots of radial failure and dose-area product for the comparison of the left radial access with the right radial access.

Overall effect estimates of the fluoroscopy time (seconds), contrast use (mL), catheter number and procedure time (minutes) are shown in Figure 2, and that of the radial failure of cardiac catheterization and dose-area product are shown in Figure 3.

**Figure 4:**
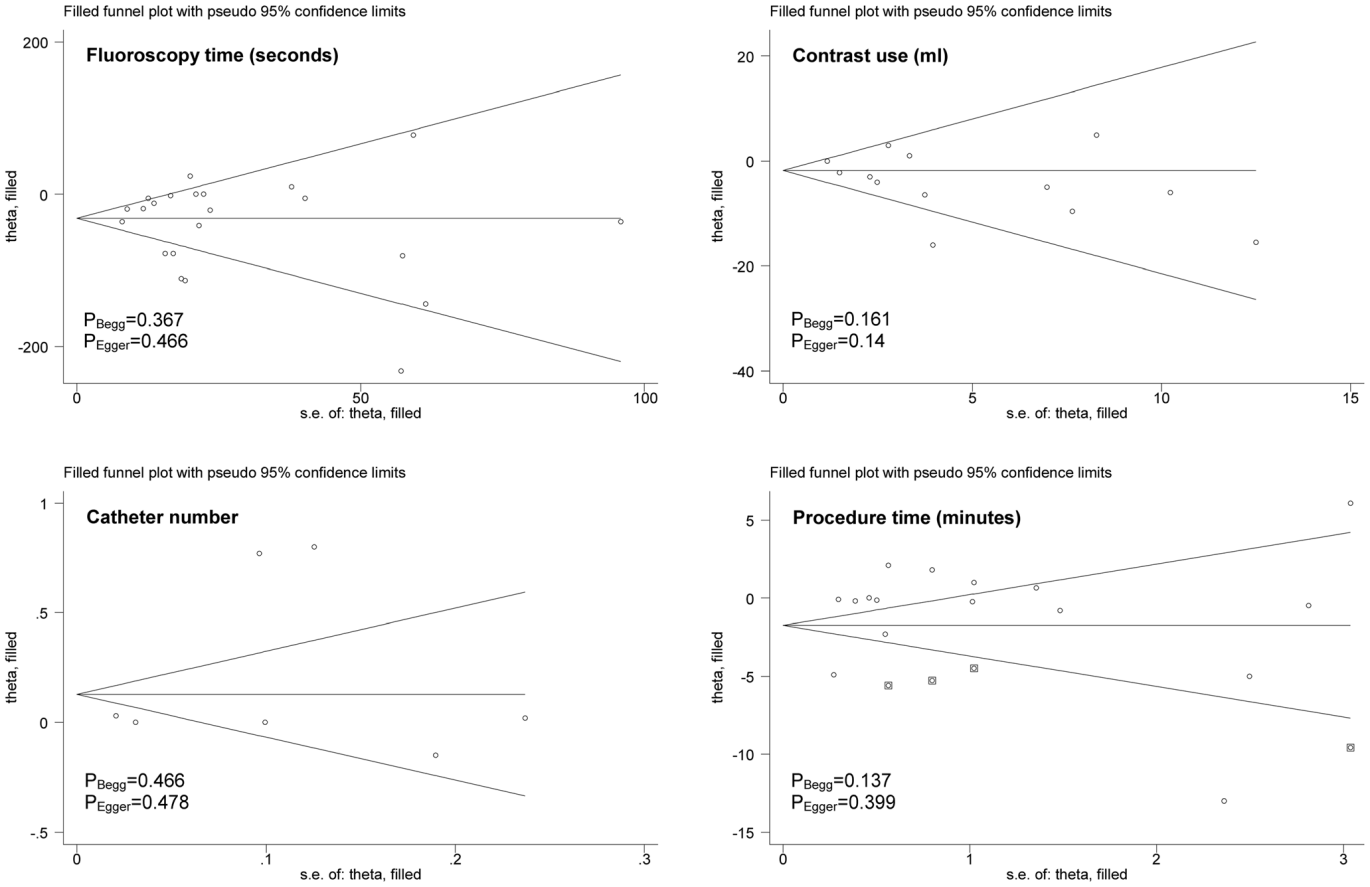
Filled funnel plots of fluoroscopy time, contrast use, catheter number, and procedure time for the comparison of the left radial access with the right radial access.

Analyses of the full data set indicated significant reductions in fluoroscopy time (WMD: –36.18; 95% CI: –53.28 to –18.53; P<0.0005) and contrast use (WMD: –2.88; 95% CI: –5.41 to –0.34; P = 0.026) in patients with the left radial access compared to those with the right radial access. The procedure time was slightly lower (WMD: –0.82; 95% CI: –2.23 to 0.6; P = 0.257) in patients with the left radial access than with the right radial access. In contrast, there was a marginally increased trend for the number of catheters in patients with the left radial access (WMD: 0.24; 95% CI: –0.02 to 0.51; P = 0.071). There was strong evidence of heterogeneity between trials for the aforementioned four overall comparisons (Figure 2). Further evidence of selective publication indicated that there was no missing trial required to make the funnel plot symmetrical for all comparisons except for the procedure time (4 missing trials were required) (Figure 4). As reflected by the Begg’s and Egger’s tests, there was low probability of publication bias for the four overall comparisons (Figure 4).

Moreover, pooling the results of all qualified trials found that the failure rate of radial access from the left was relatively lower than that from the right (OR = 0.83; 95% CI: 0.68–1.01; P = 0.064), without evidence of heterogeneity or publication bias (Figure 3). As for the dose-area product, the effect estimates between the left and the right radial accesses were comparable with strong evidence of heterogeneity.

### Subgroup analyses

Subgroup analyses by the ethnicity of trial patients, the purpose of cardiac catheterization, and the identity of the operators are summarized in Table 1 for fluoroscopy time and contrast use, and in Table 2 for catheter number and procedure time.

**Table 1:** Subgroup analyses of left versus right radial approach for fluoroscopy time and contrast use.

Subgroups	Fluoroscopy time (seconds)					Contrast use (mL)				
	Studies	WMD	95% CI	P	*I* ^2^% (P)	Studies	WMD	95% CI	P	*I* ^2^% (P)
**Ethnicity**										
**Caucasian**	10	–49.59	–83.81 to –15.36	0.005	82.1 (0.000)	8	–4.37	–9.04 to 0.3	0.066	62.4 (0.009)
**Asian**	11	–31.42	–51.9 to –10.95	0.003	77.4 (0.000)	4	–1.57	–3.77 to 0.64	0.163	26.1 (0.255)
**Mixed**	1	78.0	–38.24 to 194.24	0.188	NA	1	5.0	–11.24 to 21.24	0.546	NA
**Purpose**										
**Diagnosis**	17	–33.29	–52.05 to –14.52	0.001	81.1 (0.000)	9	–1.7	–3.3 to –0.1	0.037	5.2 (0.392)
**Intervention**	4	–59.7	–128.64 to 9.3	0.09	24.5 (0.264)	2	–7.49	–19.43 to 4.45	0.219	0.0 (0.463)
**Mixed**	3	–72.45	–159.81 to 14.91	0.104	86.4 (0.001)	3	–6.19	–20.89 to 8.52	0.41	87.1 (0.000)
**Operator**										
**Expert**	7	–47.14	–86.53 to –7.76	0.019	83.7 (0.000)	7	–3.63	–8.09 to 0.83	0.111	66.8 (0.006)
**Mixed**	5	–66.31	–114.55 to –18.07	0.007	81.4 (0.000)	4	–2.06	–5.58 to 1.47	0.253	0.0 (0.717)
**NA**	11	–22.06	–45.86 to 1.74	0.069	75.5 (0.000)	3	–3.41	–11.4 to 4.57	0.402	33.8 (0.221)

*Abbreviations*: WMD, weighted mean difference; 95% CI, 95% confidence interval; NA, not available.

**Table 2:** Subgroup analyses of left versus right radial approach for catheter number and procedure time.

Subgroups	Catheter number					Procedure time (minutes)				
	Studies	WMD	95% CI	P	*I* ^2^% (P)	Studies	WMD	95% CI	P	*I* ^2^% (P)
**Ethnicity**										
**Caucasian**	6	0.07	–0.19 to 0.34	0.599	93.9 (0.000)	5	–2.33	–5.18 to 0.53	0.11	89.9 (0.000)
**Asian**	2	0.46	–0.39 to 1.32	0.286	99.7 (0.000)	10	–0.56	–2.3 to 1.18	0.528	96.4 (0.000)
**Mixed**	1	0.8	0.55 to 1.05	0.000	NA	1	6.1	0.15 to 12.06	0.045	NA
**Purpose**										
**Diagnosis**	7	0.33	0.02 to 0.63	0.037	98.6 (0.000)	12	–0.16	–1.72 to 1.41	0.843	96.1 (0.000)
**Intervention**	0	NA	NA	NA	NA	3	–2.49	–7.13 to 2.15	0.293	61.8 (0.073)
**Mixed**	2	–0.03	–0.21 to 0.14	0.714	0.0 (0.484)	2	–6.45	–18.94 to 6.04	0.312	95.9 (0.000)
**Operator**										
**Expert**	5	0.03	–0.24 to 0.3	0.846	93.8 (0.000)	5	–1.22	–3.34 to 0.9	0.259	87.0 (0.000)
**Mixed**	2	0.0	–0.06 to 0.06	1.0	0.0 (1.0)	3	–5.79	–13.86 to 2.29	0.16	93.8 (0.000)
**NA**	3	0.66	–0.35 to 0.97	0.000	85.9 (0.001)	9	–0.11	–2.32 to 2.1	0.92	96.7 (0.000)

*Abbreviations*: WMD, weighted mean difference; 95% CI, 95% confidence interval; NA, not available.

As for fluoroscopy time, significance was preserved for the comparison of the left radial access with the right radial access in patients of Caucasian descent (WMD: –49.59; 95% CI: –83.81 to –15.36; P = 0.005), in studies with diagnostic coronary procedure (WMD: –33.29; 95% CI: –52.05 to –14.52; P = 0.001), and irrespective of the involvement of operators in training. However, significant heterogeneity cannot be explained by these subgroups. In addition, there was an obvious trend of reduction in fluoroscopy time (WMD: –59.7; 95% CI: –128.64 to 9.3; P = 0.09) in studies with interventional coronary procedure, although no significance was reached, possibly due to the small sample sizes involved, and the heterogeneity between trials was absent (P = 0.264).

With regard to contrast use, there was an obvious, albeit nonsignificant, reduction in patients of Caucasian descent (WMD: –4.37; 95% CI: –9.04 to 0.3; P = 0.066), and this reduction was significant in studies with diagnostic coronary procedure (WMD: –1.7; 95% CI: –3.3 to –0.1; P = 0.037) and heterogeneity was greatly improved (P = 0.392). Still the reduction of contrast use was obvious yet nonsignificant (WMD: –7.49; 95% CI: –19.43 to 4.45; P = 0.219) in studies with interventional coronary procedure, and there was no heterogeneity (P = 0.463).

Relative to the right radial access, a marginally significant increase in catheter numbers was observed in the left radial access in studies with diagnostic coronary procedure only (WMD: 0.33; 95% CI: 0.02 to 0.63; P = 0.037), with strong evidence of heterogeneity (P<0.0005). Regarding procedure time, no statistical significance was observed for all subgroup comparisons between the left and the right radial accesses.

### Influential analyses

Influential analyses revealed that there was not an individual trial influencing the overall effect estimates significantly. After removing each trial and calculating the overall estimates for the remaining trials, the significance of the WMDs and ORs remained materially unchanged (data not shown).

### Meta-regression analyses

A set of meta-regression analyses were conducted accordingly to explore the extent to which trial-level variables explain heterogeneity among the differences of effect estimates. It is worth noting that differences in BMI explained some part of heterogeneity for the effect estimates of both fluoroscopy time (regression coefficient: 35.85; P = 0.025) and catheter number (regression coefficient: 0.35; P = 0.018) between the left and the right radial accesses. None of the other trial-level confounders contributed significantly to the changes of all examined outcomes (data not shown).

## Discussion

Via a meta-analysis of the data from 22 randomized clinical trials and on 10287 patients, we sought to compare the left with the right radial access for the diagnostic or interventional coronary procedures. The most noteworthy finding of this study was the significant reductions of fluoroscopy time and contrast use in patients with the left radial access compared to those with the right radial access. Moreover, there was an indication of lowered failure rate of radial access from the left than the right artery. Although the potential sources of heterogeneity, albeit disturbing, could not be easily eliminated, this study, to our knowledge, is so far the most comprehensive evaluation on the comparisons between the left and the right radial accesses.

Recently, Biondi-Zoccai and colleagues have meta-analyzed the data from 5 randomized trials involving 3210 patients (mainly Caucasians), and they failed to detect any significant differences between the left and the right radial accesses in overall procedural and clinical performance [3]. Given the accumulating data in recent two years and to yield more information especially in non-Caucasian patients, we therefore updated this meta-analysis, and our overall findings demonstrate that left radial access has an obvious advantage in terms of fluoroscopy time and contrast use compared with the right radial access for the diagnostic or interventional coronary procedures. Our study is more comprehensive than the study by Biondi-Zoccai and colleagues from the following three aspects [3]. First, the present study involved 10287 patients, which enabled us to have greater power to obtain a precise effect estimate. Second, we retrieved 22 qualified articles from both English and Chinese journals, rendering it possible to perform a set of subgroup analyses. Remarkably in subgroup analyses by ethnicity, the effect estimates were comparable between Caucasians and Asians for all procedural outcomes examined. Third, extending the findings by Biondi-Zoccai and colleagues [3], we additionally performed a set of meta-regression analyses, and interestingly found that BMI might be a potential source of heterogeneity between trials, which was in agreement with the claim that obese patients had a high incidence of complications at cardiac catheterization [27].

However, it is worth mentioning that although in subgroup analyses our sample size was not intended to provide significant results in studies with interventional coronary procedure, we did identify an obvious trend of reductions in both fluoroscopy time and contrast use. It is reasonable to speculate that with the increase of sample sizes, this trend will be much clear. On the other hand, we cannot exclude the possibility that there are many factors involved in the process of percutaneous coronary intervention that might have an impact on fluoroscopy time and contrast use. We agree that confirmation of our findings in a large, well-designed clinical trial is critical.

The left radial access de facto has an important anatomical advantage because of the vascular anatomy of epiaortic vessels with a more direct access to the ascending aorta [4]. In view of this advantage, it is reasonable to expect shorter fluoroscopy time from the left radial access relative to the right, which was clearly mirrored in our overall analyses, and this expectation was more evident in patients of Caucasian descent and in studies with diagnostic coronary procedure in our subgroup analyses. What’s more, in this study radial access from the left artery seemed to be more maneuverable for operators in training than that from the right artery, because fluoroscopy time was further reduced when operators in training got involved. From a clinical standpoint, this significant reduction in fluoroscopy time was reciprocally beneficial for both patients and doctors. Besides, the more favorable vascular anatomy for the left radial access will also translate into low dose of contrast use, in agreement with the findings of this study. This observation is especially important considering the fact the contrast-induced nephropathy is known to be the third leading cause of acute renal failure [28]. Furthermore, indirect evidence from our meta-regression analyses suggested a positive and significant association of BMI with both fluoroscopy time and contrast use, conforming to the concept that the procedural difficulties are heightened in obese patients because they are mostly accompanied with atherosclerosis. Therefore, it is strongly advocated to shift the conventional radial access from the right artery to the left artery mainly for the sake of the reciprocal benefits and economic savings.

Another important finding of this meta-analysis was the relatively lower rate of radial access failure from the left than from the right. The reason behind this observation was obvious, that is, radial access from the left artery is less influenced by the subclavian tortuosity compared with that from the right artery. In fact, the presence of the right subclavian artery-common brachiocephalic trunk (CBT) and the CBT-aorta bifurcations can account for tortuosity and calcifications, which might impair the procedural success from the right radial access [1], [16]. There is also evidence suggesting a double incidence of operator-reported subclavian tortuosity associated with the right radial access compared with the left radial access [4]. Moreover, the presence of subclavian tortuosity is a major issue in prolonging the length of procedure time as the catheters must be rotated to afford the S-shaped geometry of the subclavian-innominate-aorta axis, which can increase the difficulty in catheter manipulation [29]. As expected for the right radial access, we observed relatively longer procedure time in both overall and subgroup findings of this meta-analysis, albeit no statistical significance was attained. On the other hand, radial access from the left artery can permit earlier ambulation and improve patient comfort, especially for the right-handed patients. Our findings once again highlight the priority of the left radial access in routine practice of cardiac catheterization.

Despite the clear strengths of this meta-analysis including the relatively large sample size, the low probability of publication bias, and the robustness of statistical analyses, interpretation of our findings, however, should be viewed in light of several limitations. First, we only focused on the randomized trials. Although randomized trials can minimize bias and are regarded as the gold standard for quantifying effect estimates, they may not be reflective of patients treated in general clinical practice [30]. Second, the qualified trials of this meta-analysis span more than 12 years, and during this period, changes in catheters or wires may restrict the practical implementation of the integrated data and findings. Third, there was moderate to strong evidence of heterogeneity in a majority of overall and subgroup analyses, limiting the interpretation of pooled effect estimates. Last but not least, as with all meta-analyses, despite the low probability of publication bias reported in this meta-analysis, selection bias cannot be completely excluded, since we merely searched articles from English and Chinese journals and published trials. Therefore, we must hold some reservations about the generalizability of our findings until further confirmation in larger, well-designed multicenter clinical trials.

In summary, our findings demonstrate that left radial access is preferable to right radial access in terms of fluoroscopy time and contrast use for the diagnostic or interventional coronary procedures. Moreover as expected, there was an indication of lowered failure rate of radial access from the left than the right. The import of this study lies in its great shock to the concept of convenient radial access from the right artery, which is an often-overlooked critical issue but has far-reaching implications in routine clinical practice.

## Supporting Information

Table S1 **Characteristics of the study patients of all qualified randomized trials in this meta-analysis.**
(DOC)

Checklist S1 **PRISMA checklist.**
(DOC)
