# Supplementary material for: Left Radial Access Is Preferable to Right Radial Access for the Diagnostic or Interventional Coronary Procedures: A Meta-Analysis Involving 22 Randomized Clinical Trials and 10287 Patients
Source: PLoS One. 2013 Nov 5;8(11):e78499. doi: 10.1371/journal.pone.0078499 (PMC3818350; doi:10.1371/journal.pone.0078499)
Supplement: Table S1 — Characteristics of the study patients of all qualified randomized trials in this meta-analysis. (DOC) [file pone.0078499.s001.doc]

**Table S1. Characteristics of the study patients of all qualified randomized trials in this meta-analysis**

| **Author (year)** | **Ethnicity** | **Number** | **Success rate (%)** | **Age (years)** | **Males (%)** | **BMI (kg/m^2^)** | **Smoking (%)** |
| --- | --- | --- | --- | --- | --- | --- | --- |
| Louvard Y (2001) | Caucasian | 70/70 | 100/98.6 | 61.4/60.9 | 77.1/78.6 | NA | 38.6/38.6 |
| Kawashima O (2004) | Asian | 232/205 | 99.57/97.6 | 65/66 | 46/52 | 23.1/22.8 | 23/12 |
| Fernandez-Portales J (2006) | Caucasian | 171/180 | 97.78/93.57 | 65/66 | 67.4/71 | 29/28 | NA |
| Pan Y (2006) | Asian | 30/30 | 96.7/96.7 | NA | NA | NA | NA |
| Chen C (2007) (diagnosis) | Asian | 61/56 | 95.94/95.23 | NA | NA | NA | NA |
| Chen C (2007) (intervention) | Asian | 87/91 | 96.62/96.59 | NA | NA | NA | NA |
| Chen L (2008) | Asian | 98/97 | 95.92/95.88 | NA | NA | NA | NA |
| Yao Z (2008) | Asian | 353/227 | 98/94.5 | NA | NA | NA | NA |
| Santas E (2009) | Caucasian | 335/335 | 80/82 | 66/66 | 69/70 | NA | 27/25 |
| He J (2010) | Asian | 100/100 | 97/96 | NA | NA | NA | NA |
| Wu W (2010) (diagnosis) | Asian | 38/38 | 97.4/94.7 | 57.7/58.1 | 66/71 | NA | NA |
| Wu W (2010) (intervention) | Asian | 38/38 | 97.4/94.7 | 57.7/58.1 | 66/71 | NA | NA |
| Xia F (2010) | Asian | 456/452 | 97.8/96.9 | 64.1/63.8 | 60/60 | NA | 46.9/48.5 |
| Kanei Y (2011) (total) | Mixed | 92/101 | 99/97 | 66/64 | 59/46 | 28/30 | 14/16 |
| Kanei Y (2011) (diagnosis) | Mixed | 68/82 | NA | 66/64 | 59/46 | 28/30 | 14/16 |
| Kanei Y (2011) (intervention) | Mixed | 24/19 | NA | 66/64 | 59/46 | 28/30 | 14/16 |
| Sciahbasi A (2011) (diagnosis) | Caucasian | 426/426 | 98.83/99.22 | 66.4/65.7 | 68/68 | 28/27 | NA |
| Sciahbasi A (2011) (intervention) | Caucasian | 344/344 | NA | 66.4/65.7 | 68/68 | 28/27 | NA |
| Jiang S (2011) | Asian | 558/398 | 91.2/89 | NA | NA | NA | NA |
| Dominici M (2012) | Caucasian | 612/420 | 96.1/95.5 | 69/68 | 66/64 | 27/27 | 10.7/11.8 |
| Freixa X (2012) | Caucasian | 50/50 | 90/96 | 82.2/82.9 | 58/60 | 25.9/26.6 | 40/32 |
| Norgaz T (2012) | Asian | 500/500 | 94/93.8 | 60.2/59.8 | 63/64 | 27.5/27.6 | 29/28 |
| Pacchioni A (2012) | Caucasian | 20/20 | 100/100 | 66.7/64.5 | 60/60 | 26/26.4 | 30/40 |
| Pelliccia F (2012) | Caucasian | 205/304 | 99/97 | 66/66 | 71/78 | 28/27 | 16/12 |
| Pelliccia F (2012) (expert) | Caucasian | 94/114 | 95/98 | 66/66 | 71/78 | 28/27 | 16/12 |
| Nie B (2012) | Asian | 231/232 | 98.3/97.4 | 58/58 | 60.6/62.1 | NA | 29/27.2 |
| Zhou Y (2012) | Asian | 44/85 | 97.7/95.3 | NA | NA | NA | NA |
| Dominici M (2013) | Caucasian | 204/209 | NA | 68/68 | 69/67 | 28/30 | 20/15 |

**Supplementary Table S1. (Continued)**

| **Author (year)** | **Hypertension (%)** | **Diabetes (%)** | **Dyslipidemia (%)** | **Cr (mg/dL)** | **PTCA (%)** | **MI (%)** |
| --- | --- | --- | --- | --- | --- | --- |
| Louvard Y (2001) | 42.9/47.1 | 12.9/14.3 | 58.6/62.9 | NA | 17.1/24.3 | 12.9/12.9 |
| Kawashima O (2004) | 58/61 | 33/13 | 33/28 | NA | NA | NA |
| Fernandez-Portales J (2006) | 61/56.8 | 33/34 | NA | NA | NA | NA |
| Pan Y (2006) | NA | NA | NA | NA | NA | NA |
| Chen C (2007) (diagnosis) | NA | NA | NA | NA | NA | NA |
| Chen C (2007) (intervention) | NA | NA | NA | NA | NA | NA |
| Chen L (2008) | NA | NA | NA | NA | NA | NA |
| Yao Z (2008) | NA | NA | NA | NA | NA | NA |
| Santas E (2009) | 66/69 | 35/35 | 59/62 | NA | NA | NA |
| He J (2010) | NA | NA | NA | NA | NA | NA |
| Wu W (2010) (diagnosis) | 39.47/31.58 | 18.4/23.7 | 18.4/29.0 | NA | NA | NA |
| Wu W (2010) (intervention) | 39.47/31.58 | 18.4/23.7 | 18.4/29.0 | NA | NA | NA |
| Xia F (2010) | 61.84/60.4 | 8.3/7.7 | 43.4/41.4 | NA | NA | NA |
| Kanei Y (2011) (total) | 79/82 | 41/34 | 67/62 | 1/0.9 | 27/17 | NA |
| Kanei Y (2011) (diagnosis) | 79/82 | 41/34 | 67/62 | 1/0.9 | 27/17 | NA |
| Kanei Y (2011) (intervention) | 79/82 | 41/34 | 67/62 | 1/0.9 | 27/17 | NA |
| Sciahbasi A (2011) (diagnosis) | 68/70 | 31/27 | NA | 1.04/1.05 | NA | 45/47 |
| Sciahbasi A (2011) (intervention) | 68/70 | 31/27 | NA | 1.04/1.05 | NA | 45/47 |
| Jiang S (2011) | NA | NA | NA | NA | NA | NA |
| Dominici M (2012) | 64.5/63.3 | 24.5/23.6 | 43.7/44.5 | NA | 22.4/20 | 14.4/15 |
| Freixa X (2012) | 92/80 | 13/17 | 30/29 | 1.13/1.08 | NA | 40/42 |
| Norgaz T (2012) | 57/54 | 30/27 | NA | 0.88/0.87 | NA | NA |
| Pacchioni A (2012) | 80/70 | 25/15 | 60/80 | NA | 50/45 | 25/20 |
| Pelliccia F (2012) | NA | 24/18 | NA | NA | 27/21 | 8/9 |
| Pelliccia F (2012) (expert) | NA | 24/18 | NA | NA | 27/21 | 8/9 |
| Nie B (2012) | 61/58.2 | 25.1/26.3 | 35.9/37.5 | NA | NA | NA |
| Zhou Y (2012) | NA | NA | NA | NA | NA | NA |
| Dominici M (2013) | 67/70 | 18/14 | 49/55 | NA | NA | NA |

*Abbreviations*: BMI, body mass index; Cr, creatinine; PTCA, percutaneous transluminal coronary angioplasty; MI, myocardial infarction; NA, not available. Data are expressed as left radial approach/right radial approach for numerical variables.
